# A novel facile synthesis and characterization of molybdenum nanowires

**DOI:** 10.1186/1556-276X-7-567

**Published:** 2012-10-13

**Authors:** Andrej Kovic, Andrej Znidarsic, Adolf Jesih, Ales Mrzel, Miran Gaberscek, Abdou Hassanien

**Affiliations:** 1 Jozef Stefan Institute, Jamova 39, Ljubljana, 1000, Slovenia; 2 National Institute of Chemistry, Hajdrihova 19, Ljubljana, 1000, Slovenia; 3 Electronics and Photonics Research Institute, AIST, Tsukuba, Ibaraki, 305-8568, Japan

**Keywords:** Mo nanowires, Hydrogenation, Gas phase reduction, Noncatalytic synthesis

## Abstract

We describe a straightforward technique to synthesize pure Mo nanowires (NWs) from Mo_6_S_*y*_I_*z*_ (8,2 <*y* + *z* ≤ 10) NWs as precursor templates. The structural transformations occur when Mo_6_S_*y*_I_*z*_ NWs are annealed in Ar/H_2_ mixture leading to the formation of pure Mo NWs with similar structures as initial morphologies. Detailed microscopic characterizations show that large diameters (>15 nm) Mo NWs are highly porous, while small diameters (<7 nm) are made of solid nanocrystalline grains. We find NW of diameter 4 nm can carry up to 30 μA current without suffering structural degradation. Moreover, NWs can be elastically deformed over several cycles without signs of plastic deformation.

## Background

Synthesis and characterization of nanostructured materials have been a major area of research activities in the last two decades. Nanowires (NWs), nanorods, and nanobelts constitute an important class of 1D nanostructures which provide models to study the relationship between electrical transport, optical, and other properties with dimensionality and size confinements. Investigation of these nanomaterials has attracted much attention due to their wide range of potential applications in areas such as nanoscale circuitry linkages, field electron emitters, nanosensors, and magnetic devices [1]. An important and promising method for preparing 1D nanomaterials is by using existing 1D nanostructures as templates via reactions such as metal to oxide with O_2_, oxide to metal with H_2_[2], metal or oxide to sulfide with H_2_S, carbon nanotubes to carbide with a vapor of metal oxide or halide [3], and copper oxide to copper [4]. In particular, this approach exhibits good advantages when some 1D nanostructures might be difficult or impossible to synthesize directly. Metallic molybdenum is widely used in alloy, electrode, metal particle-toughened ceramic matrix composites, and catalysts, etc. [5-8]. There are several ways in which Mo NWs have been grown successfully: e.g., large-area-aligned Mo NWs synthesized by high temperature chemical vapor deposition for application as electron emitter, [9] millimeter-scale length Mo NWs fabricated by electrochemical step edge decoration [2,10], and atomic-scale Mo NWs grown inside double-walled carbon nanotubes as templates [11]. Joule heating of Mo_6_S_3_I_6_ nanowires also causes transformation into Mo NW via thermal decomposition. The obtained NWs had 2-3 orders of conductivity higher than the starting material [12]. Nanosized molybdenum materials have received much research and industrial attention because of their unique physico-chemical properties compared with the properties of corresponding materials with larger grains including better catalytic activity and selectivity for hydrogenation [13]. Moreover, oriented mesoporous MoO_3_ thin films have been tested for battery application due to ease with which lithium ions can be stored in their van der Waals gaps [14]. Increasing porosity and surface area are greatly desirable to enhance the faradaic capacitance and therefore would increase charge storage capacity. Herein, we present a novel and efficient route to the synthesis of various morphologies of Mo NW using hydrogenation of bundles of Mo_6_S_*y*_I_*z*_ NWs as a precursor material. The method is fairly facile, scalable, and offers precise control over morphology and orientation of the end-products. Moreover, hydrogenation of large bundles (of diameters >15 nm) results in highly porous Mo NWs. These NWs are a promising material in a wide range of different applications ranging from solid state ionics, catalysts, nanoelectronic interconnects, flat panel field-emission devices, as well as fillers in composite materials. Small diameter Mo NWs (<7 nm) are made of nanocrystalline grains and can be produced by selecting similar diameters of the parent precursor. Their current-voltage (*I-V*) characteristics display metallic behavior with currents up to approximately 30 μA, for 4 nm NW, before breakdown. Such a high current density makes these NW suitable as inner interconnects in nanoelectronics.

## Methods

The precursor crystals and synthesized products have been studied by high-resolution 200 keV JEOL 2010F (JEOL Ltd., Tokyo, Japan) field-emission transmission electron microscopes (HRTEM), 80 KeV JEOL JEM-2200FS double Cs-corrector TEM and scanning electron microscope FE-SEM, Supra 35 VP. Samples were monitored by X-ray powder diffraction (XRD) using a diffractometer Bruker AXS D4 Endeavor (Bruker Corporation, Karlsruhe, Germany) with Cu-Kα1 radiation and Sol-X energy dispersive detector within the angular range 2 Θ from 6° to 73° with a step size of 0.04° and a collection time of 3 or 4 s. The samples were rotated during measurements by 6 rpm. Electrical properties of several individual Mo NWs were tested under Ar environment using a custom-built conducting atomic force microscopy (AFM) system. Lateral and vertical manipulations were achieved by constant force ranges between 0.2 to1 nN.

**Figure 1:**
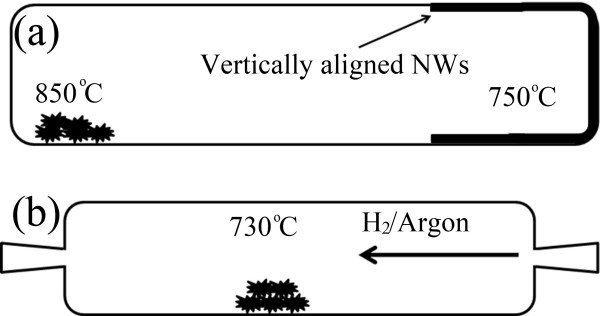
**Schematic illustration for the refinement (a) and hydrogenation (b) processes of Mo**_**6**_**S**_***y***_**I**_***z***_**NWs templates.**

There is only one step of hydrogenation process to synthesize highly porous or solid Mo NWs from their parent templates (Mo_6_S_*y*_I_*z*_ NWs). The morphology of the templates is very critical to the structural properties of the final products. For this reason, templates with various morphologies have been selectively grown by controlling the growth conditions and stoichiometry of the elements to tailor the structure of the Mo NWs. The templates are fabricated directly from the elements, with a general molar ratio of 6:*y*:*z* (8,2 <*y* + *z* ≤ 10) for Mo, S, and I, respectively. The reaction is performed at 1,045°C in a sealed quartz ampoule for 3 to 4 days after which the samples are allowed to cool spontaneously to room temperature. The process leads to the formation of Mo_6_S_*y*_I_*z*_ NWs with an average length of a few millimeters and diameter of about 500 nm. In order to reduce their diameter (<50 nm), the as-synthesized material is collected and further annealed in two zones of furnace at 850°C and 750°C (hot and cold zones, respectively). This final step yields two separate and different morphologies: a hedgehog-like morphology in the hot zone and vertically aligned NWs which are transported to the cold zone. The reduction of Mo_6_S_*y*_I_*z*_ NWs was performed by hydrogenation at 730°C in a constant flow of H_2_/Ar gas mixture. A schematic illustration of the hydrogenation setup is shown if Figure 1. In a typical synthesis process, a quartz tube with a crucible containing 100 mg of Mo_6_S_*y*_I_*z*_ NWs was initially purged with argon to remove the physisorbed gas contaminants. After several cycles of purging, a mixture of 70% of argon and 30% of H_2_ was introduced at a flow rate of 30 cm^3^/min. The quartz tube was carefully introduced into one-zone oven and gradually annealed from room temperature at a constant rate of 5°C/min till the temperature reached 730°C. With a continuous flow of H_2_/Ar mixture, the sample was annealed for 2 h before allowing it to spontaneously cool down to room temperature. It is crucial to maintain a continuous flow of gas mixture during the whole process to ensure efficient hydrogenation and prevent Mo NW from being oxidized. In a typical synthesis, several hundreds of milligrams can be obtained. Finally, the material was collected and kept in inert atmosphere (<1 ppm O_2_) before being analyzed by various microscopic techniques.

## Results and discussion

**Figure 2:**
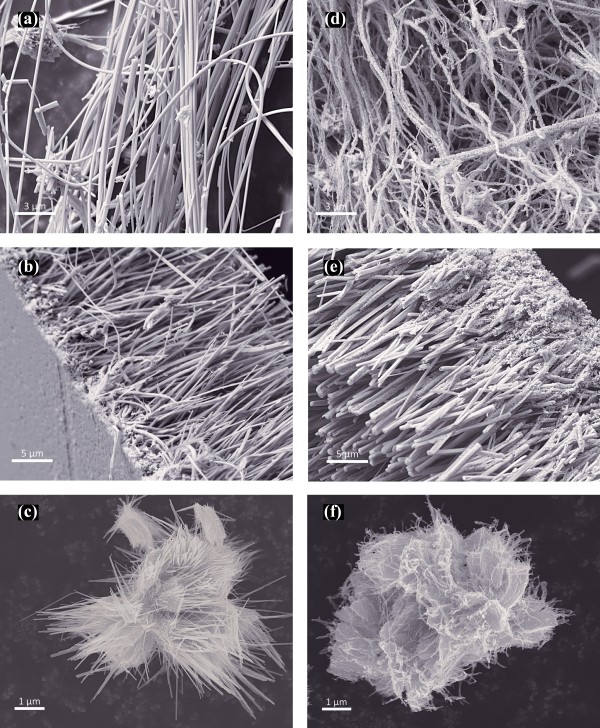
**SEM images showing several morphologies of as-synthesized MoSI NWs templates.** Left panel (**a, b, c**) and their corresponding structures after hydrogenation to produced Mo NWs at the right panel (**d, e, f**).

Typical SEM images of three different morphologies of the as-synthesized Mo_6_S_*y*_I_*z*_ (8,2 <*y* + *z* ≤ 10) NWs are shown in the left panel of Figure 2. The NWs in Figure 2a are nonaligned Mo_6_S_2_I_8_ that have a wide range of diameters and lengths. Aligned NWs can be easily produced by annealing nonaligned NWs in vacuum using two zones of temperature gradient method (with the hot zone kept at 850°C and the cold zone at 750°C). This method produces two different morphologies: one is transported to the cold zone by gas to solid condensation (aligned NWs materials) and the other remains in the hot zone. Figure 2b shows a typical example of such aligned NWs with partially uniform diameter and size. The bundles have a typical diameter up to a few hundreds of nanometers and similar length of around 20 μm. The as-grown material has a high density of quasi-aligned nanowires uniformly distributed over the entire substrate. The substrate is made of MoI_2_ materials which had been transported prior to the NWs nucleation. The formation of such substrate on top of the quartz is the key for leading to vertically aligned NW growth. The physical contact between the bundles and the MoI_2_ foil is stable enough to form free-standing film with a surface up to several squared centimeters. Furthermore, materials can easily be peeled off from the quartz substrate for further analysis. The hot zone materials are oriented in a hedgehog-like morphology with 100 nm in diameter and up to 20 μm in length (Figure 2c). Such morphology makes the hydrogenation process more efficient as it provides easy access for H_2_ to react with S and I sites. SEM images after the hydrogenation process of the three different morphologies are shown in the right panel of Figure 2. The images show that the general morphology is not altered after the hydrogenation process; however, significant pore formation is already visible, especially for the large diameter NWs.

**Figure 3:**
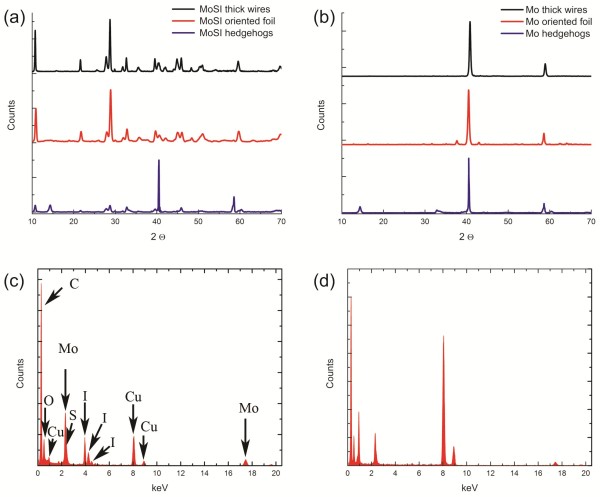
**X-ray diffraction pattern and EDS spectrum for MoSI NWs and hydrogenated samples.** (**a**) X-ray diffraction pattern for MoSI of different morphology. (**b**) X-ray diffraction pattern for hydrogenated samples in (a). (**c**) Typical example of EDS spectrum for MoSI NW. (**d**) EDS spectrum after hydrogenation shows clearly the absence of I and S peaks indicating transformation to Mo NW.

The purity of the MoSI NWs and Mo NWs obtained after the transformation was examined by XRD and energy dispersive X-ray spectroscopy (EDS) measurements. The XRD spectrum of the as-grown material is shown in Figure 3a. All reflections of the XRD spectrum are identical to MoSI NWs reported elsewhere. The spectrum can be readily indexed to a hexagonal lattice with parameters *a* = 1.6405 nm and *c* = 1.1952 nm. The absence of any impurity peaks in the XRD pattern indicates that vapor solid growth is complete and consequently, the crystalline by-products are high-purity MoSI NWs [15]. The highest peaks in the XRD pattern of the oriented NWs are coinciding with the features of Mo_6_S_3_I_6_ NWs, suggesting that such species dominate the structure of the by-product. Typical EDS spectra of MoSI NWs, presented in Figure 3c, indicate the presence of I and S together with Mo atoms. Generally speaking, we found close similarities of electron and X-ray diffraction patterns of all starting materials, indicating that these NWs have similar skeletal structures. They differ only in the site occupation of S and I atoms. After the reduction process, both XRD and EDS spectra (Figure 3b,d) show a complete absence of I and S peaks, indicating a complete transformation to pure Mo NWs.

**Figure 4:**
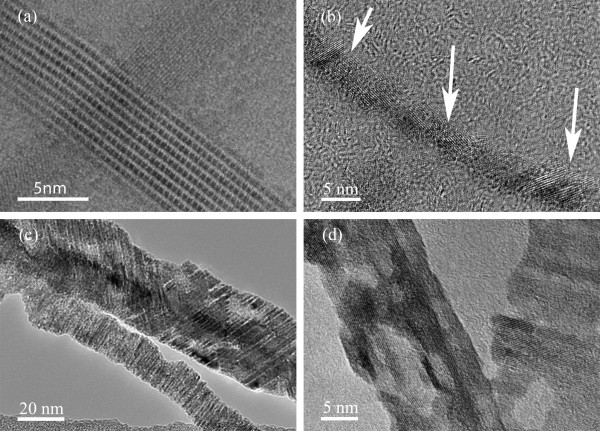
**TEM image of Mo NW and bundles.** (**a**) TEM of small MoSI NW bundle. (**b**) High resolution TEM image of Mo NW with a diameter of approximately 5 nm. The arrows point at the grain boundaries where NW can be flexible to elastic structural deformation (**c**) and (**d**) show TEM images for large diameter Mo NWs (>15 nm) with significant surface roughness. The pore formation is clearly visible as hollow voids of various sizes that are incorporated in the bulk and the surface structures.

If the hydrogenation process is performed at around 730°C, large diameter Mo NWs can be produced due to coalescence of the templates’ NW bundles. These NWs are synthesized selectively by using large bundle of Mo_6_S_2_I_8_ NWs as a precursor template. As seen in SEM (Figure 2a), the NWs have diameters up to 500 nm and can be up to several millimeters in length. The general morphology of the large diameter Mo NWs is similar to their parent NWs albeit with significant surface roughness. Interestingly, these NWs are highly porous which renders them to have a high surface to volume ratio. Such unique structural properties allow ultra-high capacitance, making them a useful material for charge storage. The pore formation is due to the fast removal of I and S atoms during the hydrogenation process, leaving voids during the subsequent crystallization of Mo atoms into small grains of sizes 3 to 50 nm (see Figure 4c,d). The shape and size of these pores are related to the kinetics of I and S removal which in turn depends on the temperature at which the hydrogenation process is performed. At the temperature of 730°C, the wires show more homogenous pores with an average size 20 nm. In NWs of diameters below 7 nm, we did not see any pore at all, suggesting that diffusion of Mo atoms is fast enough to form nanocrystalline grains that are in contact with one another. Some of these NWs can be as small as 4 nm and are stable under prolonged AFM and TEM imaging. They are also flexible to lateral manipulation; however, an onset of a 3-nN vertical force breaks them. This can be understood from the high magnification TEM image in Figure 4b which shows that the grains are stacked along the NW axis. Bending of the NW occurs right at the grains boundaries, indicating that they are held together by weak forces. This explains the resiliency of NWs to withstand several cycles of lateral deformation. Small diameter Mo NWs can be selectively grown by choosing the proper morphology of the starting MoSI templates. Although the stoichiometry criterion of the template is somewhat relaxed, the physical separation of NWs into individual or small bundles is a prerequisite for the successful synthesis of small diameter Mo NWs. For this reason, small diameter Mo NWs cannot be synthesized from the templates that form large bundles such as transported Mo_6_S_2_I_8_ NWs. The hedgehog-like morphology of hot zone materials is more suitable for use as small diameter Mo NW templates as it contains mostly small bundles and individual NW that are detached from each other. This also enhances the kinetics of the hydrogenation process due to a large access to the reactive surface area.

**Figure 5:**
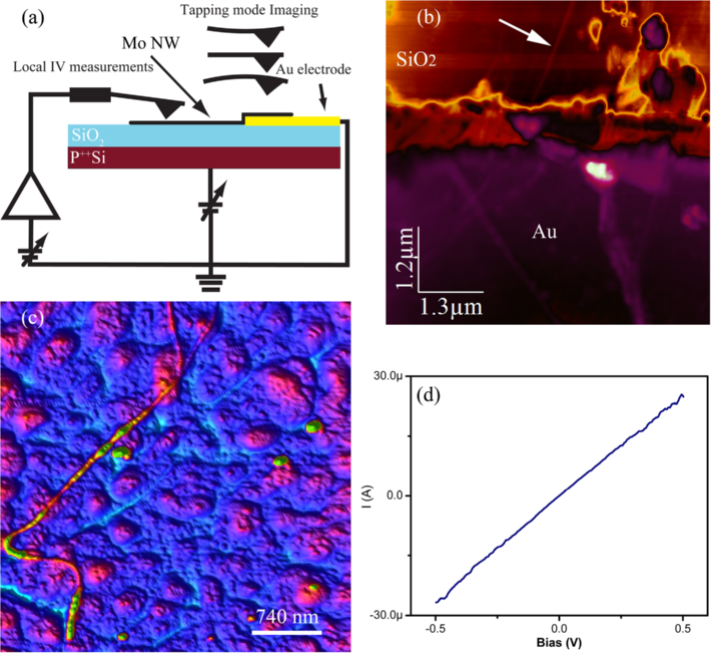
**NW layout for conductivity measurement; tapping mode, AFM image and *I-V *characteristics of manipulated Mo NW.** (**a**) Schematic of individual Mo NW layout for conductivity measurement. (**b**) Tapping mode AFM topographic image of a 4 nm Mo NW device (arrow points at the selected NW for *I-V* measurements). (**c**) AFM image of manipulated Mo NW showing its resiliency to plastic deformation. (**d**) *I-V* characteristics of 4 nm Mo NW shown in (b) exhibiting low two-terminal resistance. The NW shows a metallic conduction with a resistivity of only 2.5 larger than the Mo bulk resistivity.

The electrical properties of individual MoSI NW were tested with conducting AFM before and after the hydrogenation process. The samples were prepared by casting a few drops of Mo NW in an acetonitrile solution on a SiO_2_ substrate with prefabricated Au/Ti electrodes. The layout is shown in Figure 5a where Mo NW are contacted from one side by Au electrode and the other side is contacted by biased AFM tip as a movable source electrode. Figure 5b shows a tapping mode AFM of Mo NWs device in transistor geometry. The AFM measurements were done at room temperature in inert atmosphere to avoid oxidation and minimize surface tension forces. Tapping mode images before and after hydrogenation show morphological features which are in agreement with the data obtained from SEM and TEM techniques. Significant differences, however, are clearly observed in both electrical as well as mechanical properties. Figure 5c is a topographic AFM image of a Mo nanowire having a diameter of approximately 4 nm. The NW can be easily elastically deformed without suffering plastic deformation. A typical example of an *I-V* characteristic for the 4-nm Mo NW is shown in Figure 5d [16]. The data were collected under a constant contact force of 0.5 nN. While MoSI NWs show very high resistance (beyond the sensitivity of our measurements), a metallic behavior is clearly observed for a NW of similar diameter after hydrogenation. Moreover, the NW remarkably sustains relatively high currents, up to approximately 30 uA, without structural degradation. The resistivity value, derived from the slope of the *I-V* characteristic, is 13.7 μohm cm. This value is only 2.56 times higher than that of the bulk value (5.34 μohm cm). A higher and size-dependent resistivity is generally expected for nanosize electrodes due to a surface scattering effect and is considered one of the major hurdles in developing nanoscale interconnects. However, the relatively low resistivity coupled with a high current density make Mo NWs viable materials especially for the inner interconnects of nanoelectronic devices. The effects of electron migration, confinement, and structural deformation on the electronic properties will be reported elsewhere.

## Conclusions

In conclusion, an efficient, low-cost and scalable method has been developed to fabricate pure Mo nanowires. At first, a selected morphology of Mo_6_S_*y*_I_*z*_ is grown directly from the elements then followed by hydrogenation at 730°C. We find that the overall morphology of the synthesized Mo nanowires shows a one-to-one correspondence with the initial parent materials. This has allowed us to synthesize a variety of Mo NWs of relatively uniform diameters and lengths, including ones with aligned nanowires on free-standing foils. Large diameter Mo nanowires (>20 nm) are highly porous and can be selectively produced by controlling the growth parameters and the stoichiometry of the starting material. Such nanostructures have a very large surface area and can be advantageous for use as a host material for Li ion batteries [14] or as fillers in composites. Oriented Mo nanowires of various sizes have also been grown directly on substrates and can be integrated into different device architectures such as field-emission devices and other nanoelectronic applications. Interestingly, narrow-diameter Mo NWs (<7 nm) are flexible, highly conductive, and carry relatively large current without suffering structural degradations.

## Competing interests

The authors declare that they have no competing interests.

## Authors’ contributions

AK, AJ, AM have performed the chemical synthesis of MoSI and Mo nanowires, SEM and TEM experiments on large diameter nanowires, AZ performed the AFM experiments, MG discussed and analyzed data. AH designed the AFM experiments, performed TEM microscopy on narrow diameters Mo nanowires, and wrote the manuscript. All authors have read and approved the final manuscript.

## Authors’ information

AK is PhD student in school of International Studies of Jozef Stefan Institute. AZ is a PhD student in Ljubljana University. AJ is a research scientist from Jozef Stefan Institute. AM is a senior research scientist from Jozef Stefan Institute. MG is a professor from Ljubljana University and AH is a senior research scientist from National Institute of Industrial Science and Technology (Japan) and currently a senior research scientist from National Institute of Chemistry.

## References

[B1] Patolsky F Weizmann Y Willner I Actin-based metallic nanowires as bio-nanotransporters Nat Mater 2004 3 692 695 10.1038/nmat1205 15359342

[B2] Zach MP Ng KH Penner RM Molybdenum nanowires by electrodeposition Science 2000 290 2120 2123 10.1126/science.290.5499.2120 11118141

[B3] Dai HJ Wong EW Lu YZ Fan SS Lieber CM Synthesis and characterization of carbide nanorods Nature 1995 375 769 772 10.1038/375769a0

[B4] Qin Y Staedler T Jiang X Preparation of aligned Cu nanowires by room-temperature reduction of CuO nanowires in electron cyclotron resonance hydrogen plasma Nanotechnology 2007 18 035608 10.1088/0957-4484/18/3/035608 19636131

[B5] Fustier G Langeron JP DE LA Bastie J Carlizza J Process for the preparation of molybdenum based alloys by sintering 1978 US Patent US41151139 Sept

[B6] Iordanova I Forcey KS Gergov B Bojinov V Characterization of flame-sprayed and plasma-sprayed pure metallic and alloyed coatings Surf CoatTechnol 1995 72 23 29 10.1016/0257-8972(94)02332-K

[B7] Wei WCJ Wang SC Cheng FH Characterization of Al2O3 composites with fine Mo particulates, I. Microstructural development Nanostruct Mater 1998 10 965 981 10.1016/S0965-9773(98)00130-5

[B8] Rankin DT Stiglich JJ Petrak DR Ruh R Hot-pressing and mechanical properties of Al_2_O_3_ with an Mo-dispersed phase J Am Ceram Soc 1971 54 277 281 10.1111/j.1151-2916.1971.tb12290.x

[B9] Zhou J Deng SZ Gong L Ding Y Chen J Huang JX Chen J Xu NS Wang ZL Growth of large-area aligned molybdenum nanowires by high temperature chemical vapor deposition: synthesis, growth mechanism, and device application J Phys Chem B 2006 110 10296 10302 16722732 10.1021/jp061213z

[B10] Zach MP Inazu K Ng KH Hemminger JC Penner RM Synthesis of molybdenum nanowires with millimeter-scale lengths using electrochemical step edge decoration Chem Mater 2002 14 3206 3216 10.1021/cm020249a

[B11] Muramatsu H Hayashi T Kim YA Shimamoto D Endo M Terrones M Dresselhaus MS Synthesis and isolation of molybdenum atomic wires Nano Lett 2008 8 237 240 10.1021/nl0725188 18069873

[B12] Hummelgard M Zhang RY Carlberg T Vengust D Dvorsek D Mihailovic D Olin H Nanowire transformation and annealing by joule heating Nanotechnology 2010 21 165704 10.1088/0957-4484/21/16/165704 20351407

[B13] Li YZ Luo GF Fan YN Chen Y A novel route to the synthesis of nanosized metallic molybdenum at moderate temperature and its catalytic properties Mater Res Bull 2004 39 195 203 10.1016/j.materresbull.2003.10.020

[B14] Brezesinski T Wang J Tolbert SH Dunn B Ordered mesoporous α-MoO3 with iso-oriented nanocrystalline walls for thin-film pseudocapacitors Nat Mater 2010 9 146 151 10.1038/nmat2612 20062048

[B15] Meden A Kodre A Gomilsek JP Arcon I Vilfan I Vrbanic D Mrzel A Mihailovic D Atomic and electronic structure of Mo6S_9−xix_ nanowires Nanotechnology 2005 16 1578 1583 10.1088/0957-4484/16/9/029

[B16] Horcas I Fernandez R Gomez-Rodriguez JM Colchero J Gomez-Herrero J Baro AM WSXM: a software for scanning probe microscopy and a tool for nanotechnology Rev Sci Instrum 2007 78 013705 10.1063/1.2432410 17503926

